# Analgesic efficacy and safety of erector spinae plane block in breast cancer surgery: a systematic review and meta-analysis

**DOI:** 10.1186/s12871-021-01277-x

**Published:** 2021-02-20

**Authors:** Ying Zhang, Tieshuai Liu, Youfa Zhou, Yijin Yu, Gang Chen

**Affiliations:** Department of Anesthesiology, Sir Run Run Shaw Hospital, School of Medicine, Zhejiang University, Hangzhou, 310020 China

**Keywords:** Breast surgery, Erector spinae plane block (ESPB), Opioid consumption, Postoperative analgesia

## Abstract

**Background:**

Surgical resection is considered to be the primary and most effective therapy for breast cancer, postoperative pain is an issue gaining significant attention. In recent years, erector spinae plane block (ESPB) has attracted much attention in postoperative analgesia, but its effectiveness is still controversial. This meta-analysis was implemented to verify the clinical analgesic efficacy and safety of erector spinae plane block in patients undergoing breast cancer surgery.

**Methods:**

We searched PubMed, EMBASE, Web of Science, the Cochrane Library and ClinicalTrials.gov for randomized controlled trials (RCTs) comparing ESPB with general anesthesia (GA) in breast cancer surgery that were published before December 25, 2020. The primary outcome was opioid consumption at the first 24 h after surgery, while secondary outcomes included pain scores at 1, 6,12 and 24 h after surgery, opioid consumption at 1, 6 and 12 h after surgery, intraoperative opioid consumption, number of patients who need for rescue analgesia, and the incidence of postoperative nausea and vomiting (PONV).

**Results:**

Eleven randomized controlled trials involving 679 patients met the study inclusion criteria and were included in this study. In comparison to GA group, the ESPB group showed a significant reduction in morphine consumption at the first 24 h after surgery by a mean difference (MD) of − 7.67 mg [95% confidence interval (CI) − 10.35 to − 5.00] (*P* <  0.01). In addition, the ESPB group showed lower pain scores than the GA group in the four time periods (1, 6, 12 and 24 h after surgery). ESPB group significantly reduce the intraoperative consumption of fentanyl, the need for postoperative rescue analgesia, and the incidence of PONV.

**Conclusions:**

Ultrasound-guided ESPB is an effective approach for reducing morphine consumption and pain intensity within the first 24 h after breast cancer surgery, compared with GA alone.

**Supplementary Information:**

The online version contains supplementary material available at 10.1186/s12871-021-01277-x.

## Background

Breast cancer is the most commonly diagnosed cancer and the leading cause of cancer-related mortality among females in more than 100 countries [1]. The traditional therapeutic approaches for breast cancer include surgery, radiation therapy, chemotherapy, Immunotherapy and endocrine therapy [2–4], surgical resection is still considered to be the primary and most effective therapy [5]. However, breast surgery is usually associated with postoperative pain with varying intensity and duration. In addition, poor management of acute postoperative pain may lead to persistent postoperative pain, also known as chronic pain [6], which affecting approximately 25 to 60% of patients [7]. Therefore, it is necessary to provide appropriate perioperative interventions to alleviate postoperative pain in such patients.

The erector spinae plane block (ESPB) is a relatively new regional blocking technique that can be utilized to reduce postoperative pain effectively in various surgical procedures such as breast, thoracic, abdominal and lumbar surgery. It was first described in 2016 by Forero, as a successful interfascial plane block for thoracic neuropathic pain [8]. Bonvicini et al. first reported a case of clinical use of ESPB for postoperative pain control after breast surgery, which promoted rapid recovery following surgery [9]. In the following two years, the application of ESPB in breast surgery has risen dramatically. Nevertheless, the effectiveness of ESPB is still controversial. Thus, a meta-analysis is conducted in this study to evaluate the efficacy and safety of ESPB in breast cancer surgery. We included randomized controlled trials (RCTs) comparing ESPB with general anesthesia (GA) alone as control in females undergoing breast cancer surgery. The primary objective of this meta-analysis was to determine if ESPB is effective for reducing morphine consumption at the first 24 h after surgery. For secondary objectives, we aimed to compare pain scores after surgery, intraoperative opioid consumption, the incidence of PONV and block-related adverse events.

## Methods

This meta-analysis was performed according to the guideline of the Preferred Reporting Items for Systematic Reviews and Meta-Analyses (PRISMA) statement [10].

### Search methods

A manual search for relevant studies was performed in PubMed, EMBASE, Web of Science, the Cochrane Library and ClinicalTrials.gov from the establishment of the database to December 25, 2020. There were no language restrictions. The search terms included a wide range of synonyms and were established using a combination of MeSH terms and free terms, including “erector spinae block, erector spinae plane block, ESP block or ESPB” and “breast surgery, breast cancer surgery or mastectomy.” The detailed search strategy used for each database are presented in Supplemental data [see Additional file 1]. In addition to the above, we also manually searched the lists of journals and references for any relevant articles to this study. This meta-analysis was based on the studies published previously, so ethical approval and patient consent were not necessary.

### Eligibility criteria

Eligibility criteria for studies are defined based on PICOS standards (participants, interventions, comparisons, outcomes and study designs).

#### Types of participants

Adult female patients aged 18–70 years with American Society of Anesthesiologists (ASA) physical status I-II and scheduled for elective surgery for breast cancer were included in this study. Patients with other surgeries would be excluded, such as breast brachytherapy, radiofrequency ablation of liver tumors, lumbar surgery and thoracoscopic surgery.

#### Types of interventions

Ultrasound (US)-guided ESPB was performed in the experimental group, and the control group was placebo or received no intervention. If the control group was included in the article which compared ESPB versus other type of nerve blocks, these articles would be included.

#### Types of comparisons

Comparisons will be made between the experimental (ESPB) group and the control (general anesthesia) group.

#### Types of outcomes

The main outcome of this meta-analysis is to compare opioid consumption at the first 24 h after surgery. For secondary objectives, we aimed to compare pain scores after surgery, intraoperative opioid consumption, the incidence of PONV and block-related adverse events.

#### Types of study designs

Randomized controlled trials (RCTs) are the only study type to be included. Case reports, reviews, editorials, and registration trials without full text will be excluded.

All articles retrieved were stored into EndNote version X9 software to remove duplicates from the initial literature search automatically. Two reviewers will independently scan the titles and abstracts of retrieved studies to identify studies that meet the inclusion criteria according to the predefined eligibility criteria. The full-text was further reviewed if decision could not be made after reading title and abstract. Disagreements between reviewers were resolved by discussion or referral to a third reviewer if necessary.

### Data extraction

Two authors independently extracted data and entered into a standard template including: first author, year of publication, types of surgery, number of patients, time of block, concentration and volume of local anesthetic, assessment of block success, intraoperative and postoperative analgesia, and the adverse events. The primary outcome variable was opioid consumption at the first 24 h after surgery, the secondary outcome variables were: pain scores at 1, 6,12 and 24 h after surgery, opioid consumption at 1, 6 and 12 h after surgery, intraoperative opioid consumption, number of patients who need for rescue analgesia, and the incidence of PONV. Whenever pain scores were reported at rest or active movement, we extracted the worst pain score at every time point as needed. We digitized the data by GetData Graph Digitizer (version2.25) if data were presented in graphical formats. When data were given as median and interquartile range, the mean and standard deviation were estimated following the approach detailed by Luo et al. [11]. If any opioids were given as analgesia, in order to standardise outcome measures, intraoperative opioid dose was converted to intravenous fentanyl equivalents (ug) and postoperative opioid dose was converted to intravenous morphine equivalents (mg) [12–14]. If separate data on PONV was reported by the study, the number of patients with nausea was extracted. Two reviewers independently extracted the research data using a standard data sheet.

### Quality assessment

The risk of bias for all included studies were assessed using the updated Cochrane RoB 2.0 tool [15]. This tool evaluated bias following five domains: (1) bias arising from the randomisation process; (2) bias due to deviations from intended interventions; (3) bias due to missing outcome data; (4) bias in measurement of the outcome; (5) bias in selection of the reported result. According to the relevant standards in the Cochrane risk-of-bias tool for randomized trials, each domain was classified as “low risk of bias,” “some concerns,” and “high risk of bias.” An overall risk of bias judgement will be made for each study according to domain-level judgements. Each domain of included studies was assessed by two reviewers independently and any disagreements were adjudicated by discussion or referral to a third reviewer if necessary.

### Statistical methods

Data analysis was performed using the Review Manager software (RevMan, version 5.3) and STATA version 12.0, and a *P* value< 0.05 was considered statistically significant. Mean differences with corresponding 95% CI were calculated for continuous data, and risk ratios (RR) combined with 95% confidence interval (95% CI) were calculated for dichotomous data. Statistical heterogeneity was estimated by the I^2^ statistic. A value of I^2^ > 50% was considered to indicate significant heterogeneity, the random-effect model would be used, otherwise a fixed-effect model was used. For the primary outcome, a sensitivity analysis was performed by leave-one-out approach to find possible the sources of heterogeneity and subgroup analysis according to different levels of risk of bias and different local anesthetics were also performed. Moreover, subgroup analysis was used to investigate pain scores at different time points (1 h, 6 h, 12 h, 24 h) after surgery. Potential publication bias was identified by the funnel plot and Egger’s test in meta-analysis that included more than nine studies.

## Results

### Results of search

**Fig. 1:**
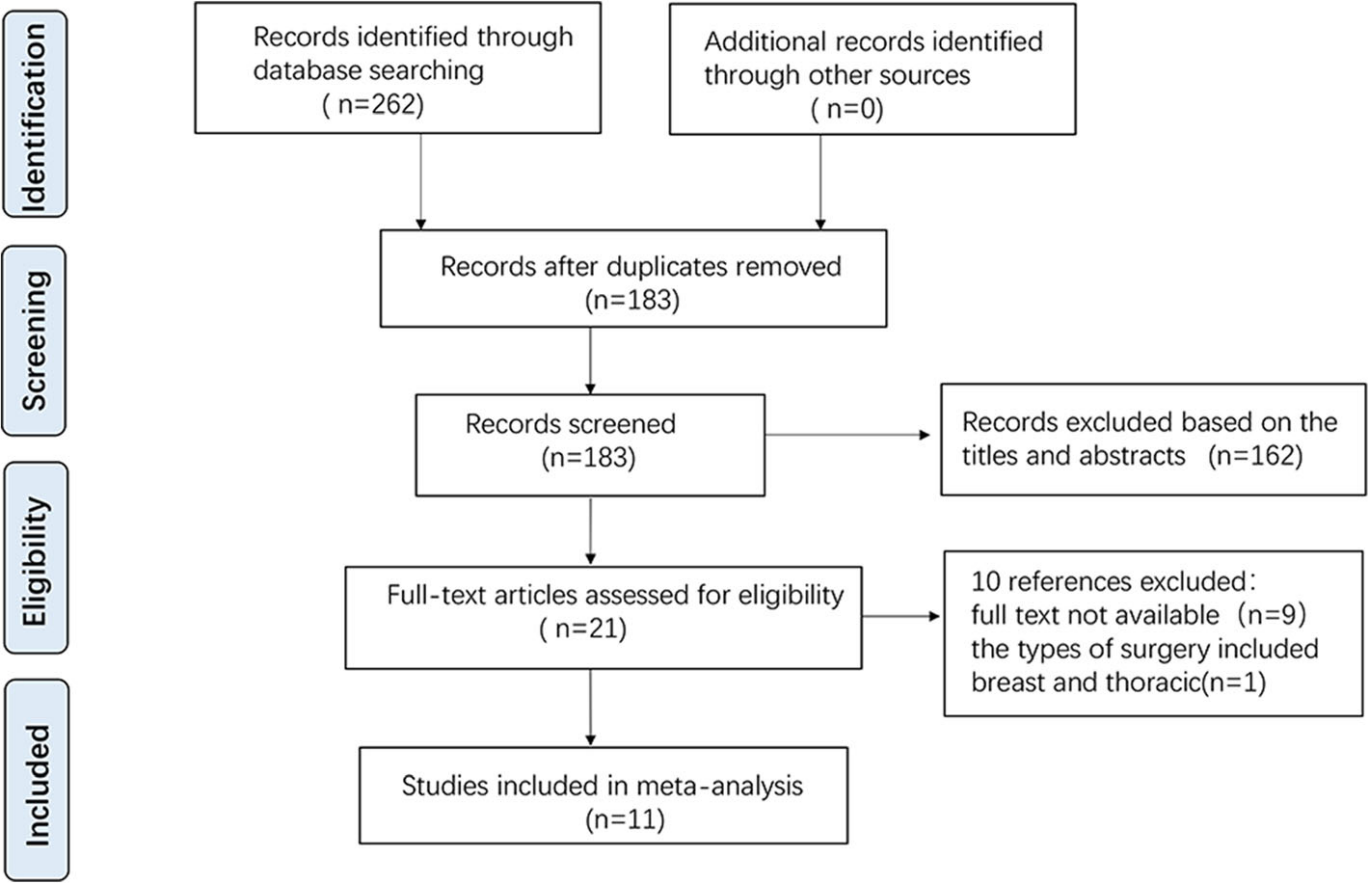
Flow diagram of the literature search and study selection

Figure 1 presents the flow diagram of the literature search and study selection. A total of 262 relevant studies were preliminarily identified after a systematic literature search and 79 of them were excluded after duplicate removing. One hundred and sixty-two of the remaining studies were further excluded after screening of titles and abstracts. The remaining 21 articles were assessed in more detail for eligibility by reading the full text. Nine studies which were registered on the International Clinical Trial Registry were excluded because the full text could not be retrieved. Another study was additionally excluded because the types of surgery included breast and thoracic. Thus, a total of 11 RCTs [16–26] with 679 patients met the inclusion criteria and were included in the final analysis.

### Description of included studies

The characteristics of the included studies are presented in Table 1. A total of 679 patients undergoing breast cancer surgery from 11 trials were collated and grouped into ESPB or only GA groups, 339 in ESPB group, and 340 in GA group. The number of patients per group in each study varied from 20 to 50. Among these 11 trials, 9 [16–22, 24, 26] of them included modified radical mastectomy, with sentinel lymph node biopsy in two trials [16, 18] and axillary lymph node dissection in three trials [16, 18, 20]. Except for the study by Elsabeeny et al. [17], the rest of the included studies showed that ESPB was performed by ultrasound before the induction of general anesthesia with or without preoperative sedation. Most trials [17–26] received a single-level technique at the T4 or T5 vertebral level, and only one trial [16] used at bi-level (T2-T4) block technique. The control group of one trial was treated with 0.9% physiological saline as sham block [26], the other control groups were received no intervention. Among these 11 trials, seven [16–19, 21, 22, 24] of them used bupivacaine, and six used bupivacaine at a concentration of 0.25% for their block. Four [20, 23, 25, 26] of them were given ropivacaine. Assessment of sensory block was performed for mapping the block area in three trials, all blocks performed in these studies were successful [23–25]. In most studies, postoperative pain was provided by patient-controlled intravenous analgesia with morphine, while three trials [17, 20, 22] used morphine and flurbiprofen axetil as single-dose rescue analgesic. There were no complications associated with nerve block reported in any trials, such as vascular puncture, pneumothorax, or local anesthesia toxicity. Two trials reported skin itch and dizziness, respectively [25, 26].

**Table 1:** The characteristics of the included studies

author/year	types of surgery	Time of block	No.	type of block and local anaesthetic	assessment of sensory Block	intraoperative analgesia	Postoperative analgesia	Complications
Aksu 2019 [16]	MRM; mastectomy+SLNB; Lumpectomy+ ALND	Before induction of GA	25	ESPB group: 0.25% bupivacaine 20 ml (10 ml for each level) at bi-level (T2-T4)	N	fentanyl 2 mg/kg, tramadol, paracetamol	morphine PCA	Not reported
			25	GA group: Received no intervention				
Elsabeeny 2020 [17]	MRM	After induction of GA	25	ESPB group: 0.25% bupivacaine 25 mL at the T5 vertebral level	N	intravenous morphine 0.1 mg/kg.	Ketorolac morphine prn	Not reported
			25	GA group: Received no intervention				
Gürkan 2018 [18]	MRM; mastectomy+SLNB; Lumpectomy+ ALND	Before induction of GA	25	ESPB group: 0.25% bupivacaine 20 ml at the T4 vertebral level	N	fentanyl 2 mg/kg, tramadol, paracetamol	morphine PCA	Not reported
			25	GA group: Received no intervention				
Gürkan 2020 [19]	MRM;Breast conserving surgery mastectomy	Before induction of GA	25	ESPB group: 0.25% bupivacaine 20 ml at T4 vertebral level.	N	fentanyl 2 mg/kg, tramadol, paracetamol	morphine PCA paracetamol 1 mg for every 6 h	Not reported
			25	GA group: Received no intervention				
He 2020 [20]	MRM ± ALND	Before induction of GA	20	ESPB group: 0.5%ropivacaine 20 ml at the vertebral T3 level	N	N/S	Flurbiprofen axetil prn	Not reported
			20	GA group: Received no intervention				
Li 2020 [21]	MRM	Before induction of GA	30	ESPB group: 0.25% bupivacaine 30 ml at T3-T5 vertebral level.	N	fentanyl	morphine PCA	Not reported
			30	GA group: Received no intervention				
Seelam 2020 [22]	MRM	Before induction of GA	50	ESPB group: 0.25% bupivacaine 30 ml T4 transverse process	N	fentanyl 1.5μg/kg	Morphine prn paracetamol 1 g TID	Not reported
			50	GA group: Received no intervention				
Sharma 2020 [23]	total mastectomy + ALND	Before induction of GA	30	ESPB group: 0.5%ropivacaine 20 ml, 0.4 mL/kg the T5 level	Y	fentanyl 1μg/kg, diclofenac 1.5 mg/kg	morphine PCA paracetamol 1 g TID	Not reported
			30	GA group: Received no intervention				
Singh 2019 [24]	MRM	Before induction of GA	20	ESPB group: 0.5% bupivacaine 20 ml at the T5 vertebral level	Y	morphine 0.1 mg/kg	morphine PCA, diclofenac 1.5 mg/kg TID	Not reported
			20	GA group: Received no intervention				
Wang 2019 [25]	radical mastectomy	Before induction of GA	50	ESPB group: 0.375%ropivacaine 20 ml at the T5 spinous process	Y	sufentanil 0.4 μg/kg	Sufentanil PCA, Flurbiprofen axetil prn	skin itch
			50	GA group: Received no intervention				
Yao 2019 [26]	MRM	Before induction of GA	39	ESPB group: 0.5%ropivacaine 25 ml at the T4 spinous process	N	Sufentanil 0.5 μg/kg	Sufentanil PCA flurbiprofen axetil 50 mg TID	Not reported
			40	GA group: 0.9% physiological saline 25 ml				

MRM: modified radical mastectomy; SLNB: sentinel lymph node biopsy; ALND: axillary lymph node dissection; N/S: not specified; N: no; Y: yes; PCA: patient-controlled analgesia; prn: as needed; ESPB: erector spinae plane block; GA: general anesthesia

### Risk of bias within studies

**Fig. 2:**
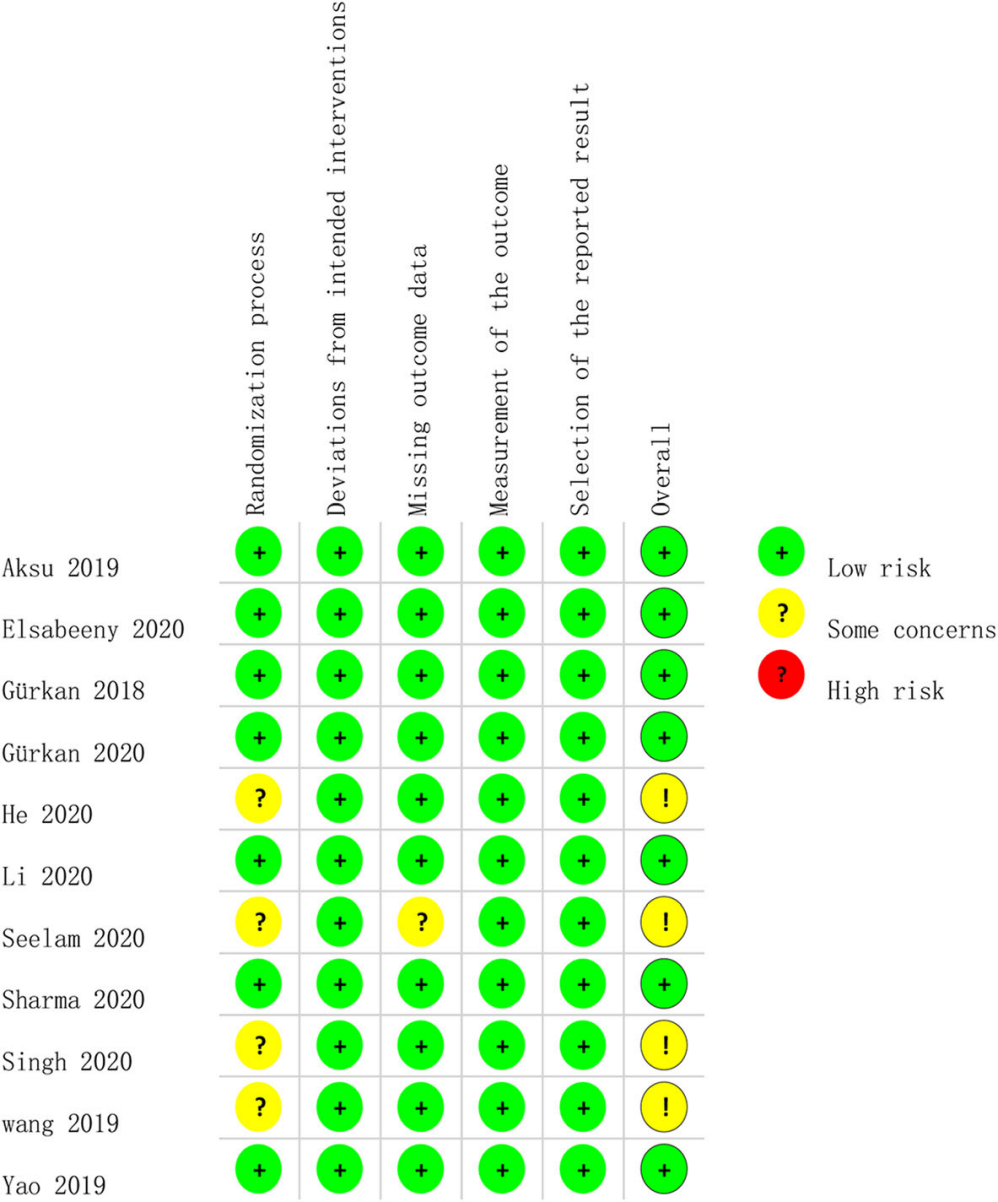
The risk of bias assessment for included studies. Green, yellow and red circles indicate low, some concerns and high risk of bias, respectively

The risk assessment of the included studies is presented in Fig. 2. All of the included studies in the analysis were random control study and the method of random allocation were clearly described, and four [20, 22, 24, 25] did not provide sufficient information about allocation concealment. One study [22] did not provide information about pain score. Overall, the quality of the included studies was good, but are defective in the area of participants and personnel blinding.

### Opioid consumption at the first 24 h after surgery

**Fig. 3:**
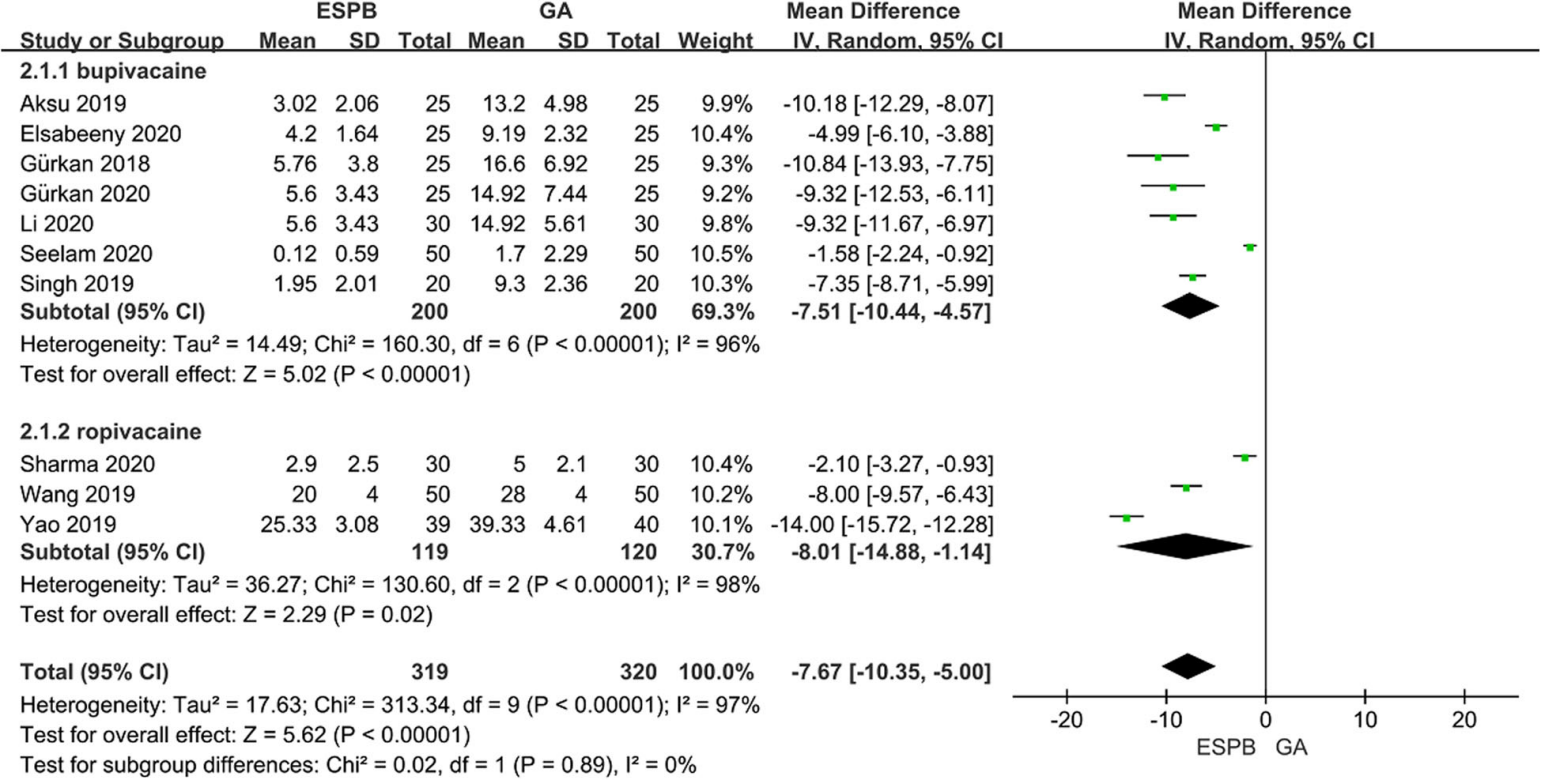
Forest plot of total opioid consumption at the first 24 h after surgery

Ten studies [16–19, 21–26] included 639 patients reported opioid consumption (converted to Intravenous morphine equivalents) at the first 24 h after surgery. Heterogeneity was detected (I^2^ = 97%) so the random effects model was used. Patients receiving ESPB showed a significant reduction in morphine consumption compared with the GA group at the first 24 h after surgery (MD: –7.67; 95%CI: − 10.35 to − 5.00; *P* <  0.01) (Fig. 3). Subgroup analysis was used to evaluate the efficacy of different local anesthetics on outcome indexes, and it was confirmed that there was a good consistency between the two local anesthetics. The results of this sensitivity analysis are summarized in Table 2. After the exclusion of any one study, the direction and magnitude of the primary outcome did not change significantly, indicating that the meta-analysis had good reliability and robustness. We further conducted a subgroup analysis of low-risk of bias studies versus some concerns of bias studies, also, there was no apparent difference between the subgroups (*P* = 0.31). [see Additional Fig.S1].

**Table 2:** Sensitivity analysis of Opioid consumption at the first 24 h after surgery

Study	statistics with study removed				
	MD	Lower limit	Upper limit	Z value	P value
Aksu 2019 [16]	−7.39	−10.18	−4.61	5.21	< 0.01
Elsabeeny 2020 [17]	−8.00	−11.14	−4.86	5.00	< 0.01
Gürkan 2018 [18]	− 7.35	− 10.13	− 4.56	5.17	< 0.01
Gürkan 2020 [19]	− 7.51	−10.32	− 4.69	5.23	< 0.01
Li 2020 [21]	− 7.49	−10.31	− 4.67	5.21	< 0.01
Seelam 2020 [22]	−8.37	− 10.93	−5.82	6.42	< 0.01
Sharma 2020 [23]	− 8.33	−11.33	− 5.32	5.44	< 0.01
Singh 2019 [24]	−7.72	− 10.68	− 4.76	5.11	< 0.01
Wang 2019 [25]	− 7.64	−10.54	− 4.74	5.17	< 0.01
Yao 2019 [26]	−6.91	−9.26	− 4.56	5.77	< 0.01

MD = mean difference

### Opioid consumption at 1,6,12 h (< 24 h) postoperatively

**Fig. 4:**
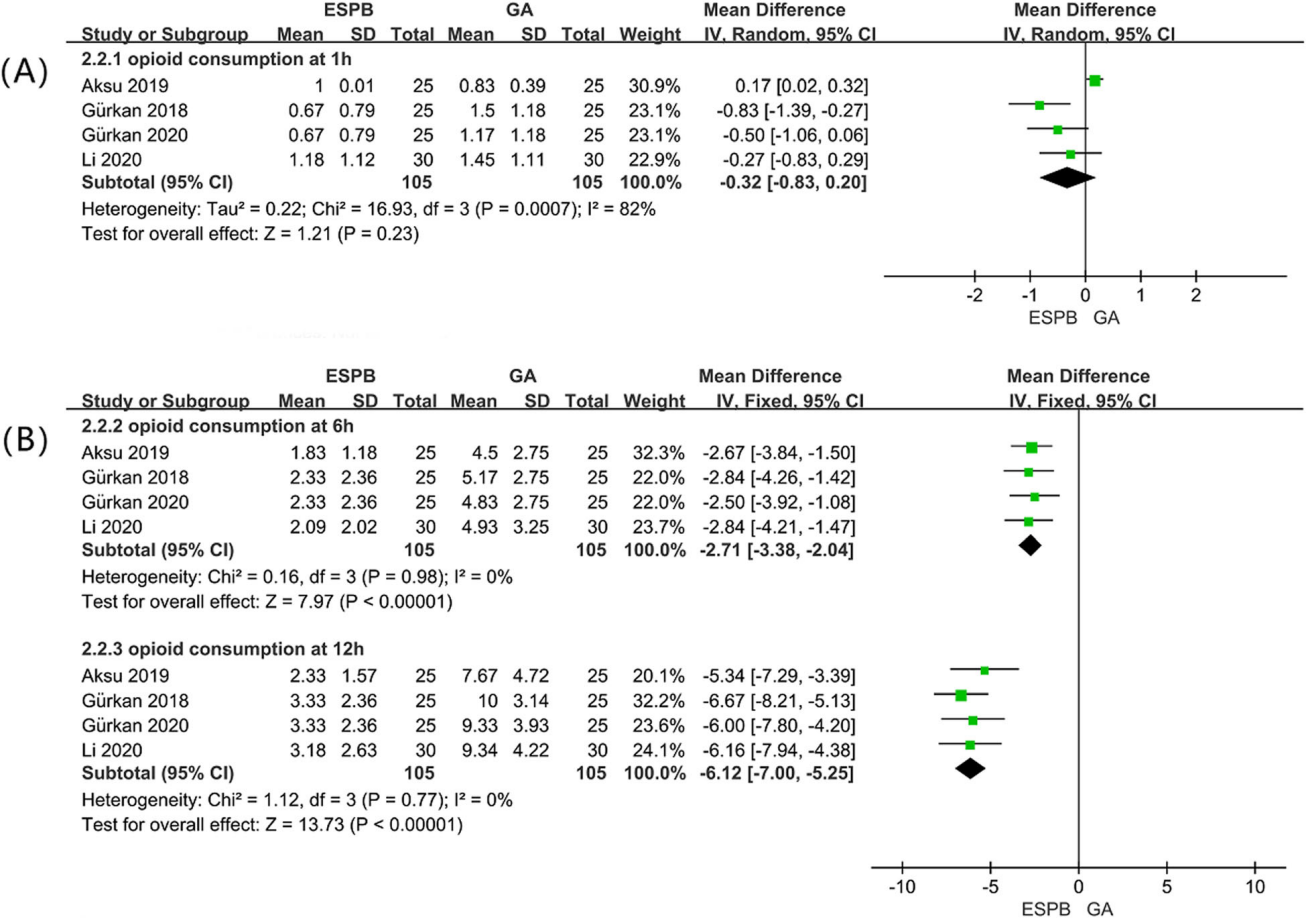
Forest plot of total opioid consumption. (A) total opioid consumption at 1 h postoperatively. (B) total opioid consumption at 6,12 h postoperatively

Four studies [16, 18, 19, 21] included 210 patients reported opioid consumption (converted to Intravenous morphine equivalents) at 1,6,12 h postoperatively. There were no statistically significant differences between the ESPB group and the GA group with regard to opioid consumption at 1 h postoperatively (MD: –0.32; 95% CI: − 0.83 to 0.20; *P* = 0.23] (Fig. 4a). Patients receiving ESPB showed a significant reduction in morphine consumption compared with the GA group at 6 and 12 h postoperatively, by a mean difference [95% CI] − 2.71 [− 3.38, − 2.04] (*P* <  0.01, I^2^ = 0%), − 6.12[− 7.00, − 5.25] (P <  0.01, I^2^ = 0%), respectively (Fig. 4b).

### Pain scores (VAS/NRS) at 1, 6, 12, and 24 h postoperatively

**Fig. 5:**
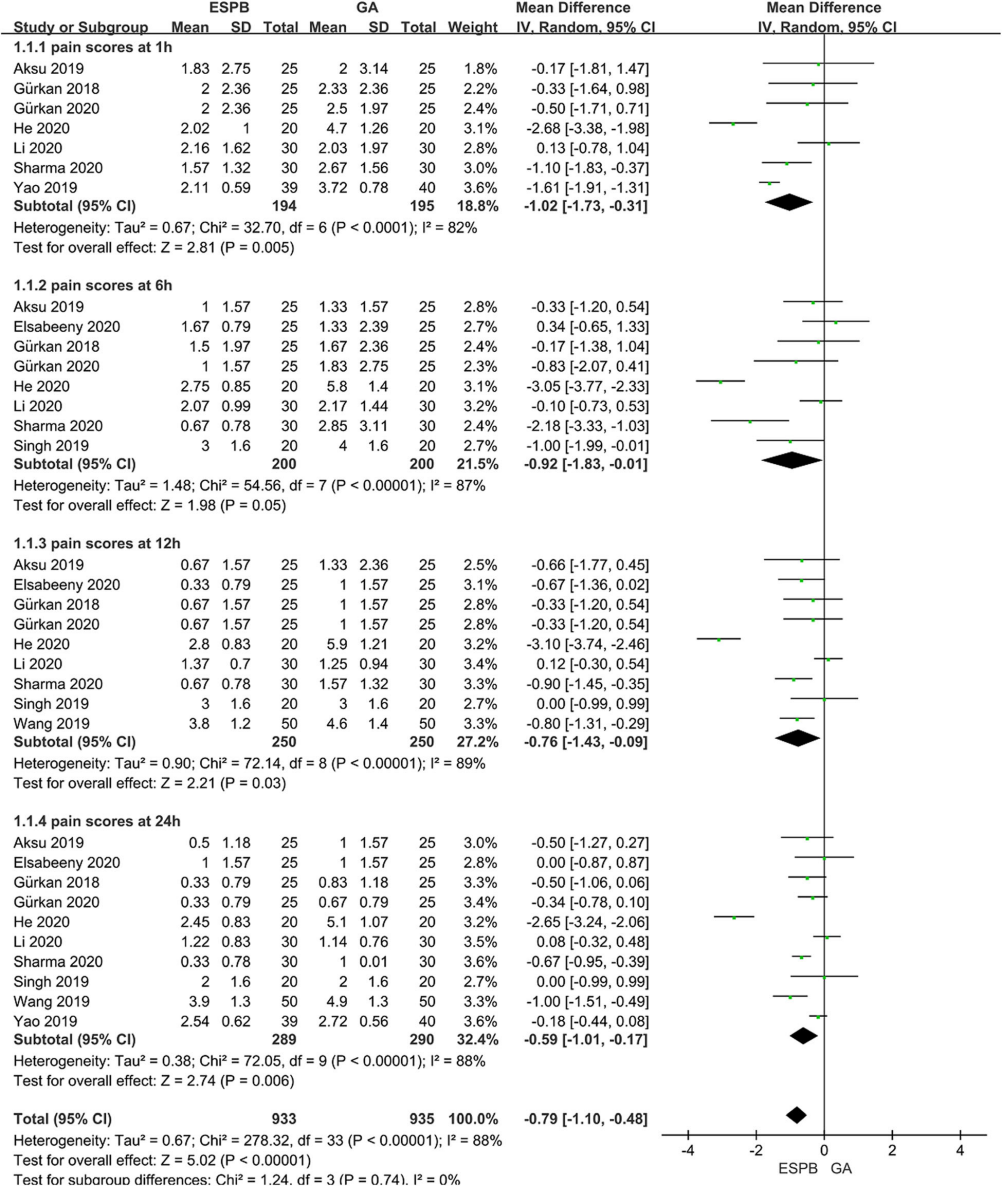
Forest plot of pain scores postoperatively

Pain scores (VAS/NRS) were significantly lower at all time-points (at 1, 6, 12, and 24 h after surgery) in patients receiving ESPB than that in the GA group, by a mean difference [95% CI] − 1.02 [− 1.73, − 0.31] (P <  0.01, I^2^ = 82%), − 0.92[− 1,83, − 0.01] (*P* = 0.05, I^2^ = 87%), − 0.76 [− 1.43, − 0.09] (*P* = 0.03, I^2^ = 89%), and − 0.59 [− 1.01, − 0.17) (P <  0.01, I^2^ = 88%), respectively (Fig. 5).

### Incidence of PONV at 24 h postoperatively

**Fig. 6:**
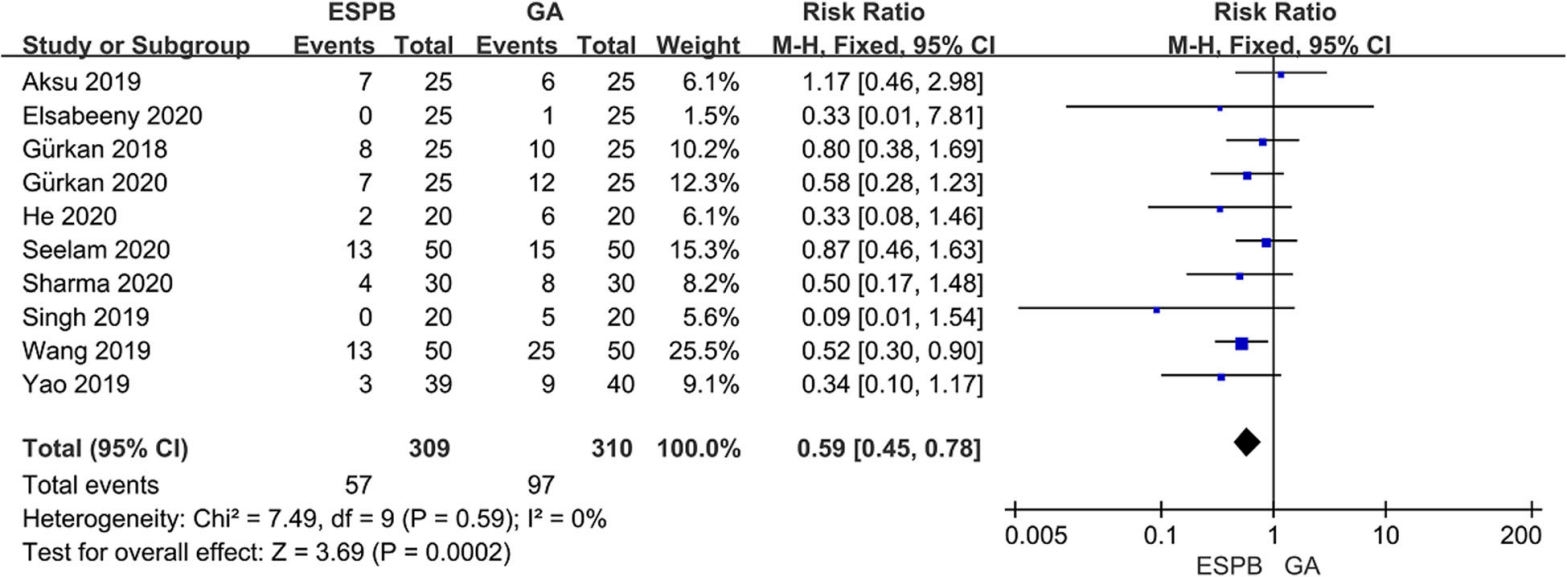
Forest plot of the incidence of postoperative nausea and vomiting (PONV)

Ten studies included 619 patients investigated incidence of PONV at 24 h postoperatively. The value of I^2^ was calculated to be 0%, the fixed-effects model was then used. The incidence of PONV was significantly lower in patients receiving ESPB than that in the GA group (RR 0.59; 95%CI 0.45 to 0.78; *p* <  0.01) (Fig. 6).

### Intraoperative opioid consumption

**Fig. 7:**
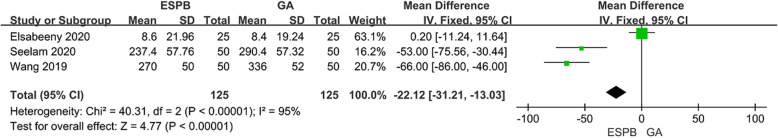
Forest plot of intraoperative opioid consumption (fentanyl equivalents)

Three studies [17, 22, 25] included 250 patients measured the intraoperative opioid consumption (converted to Intravenous fentanyl equivalents). Patients receiving ESPB showed a significant reduction in fentanyl consumption compared with the GA group (MD: –22.12; 95%CI: − 31.21 to − 13.03; *P* <  0.01) (Fig. 7).

### Postoperative rescue analgesia

**Fig. 8:**
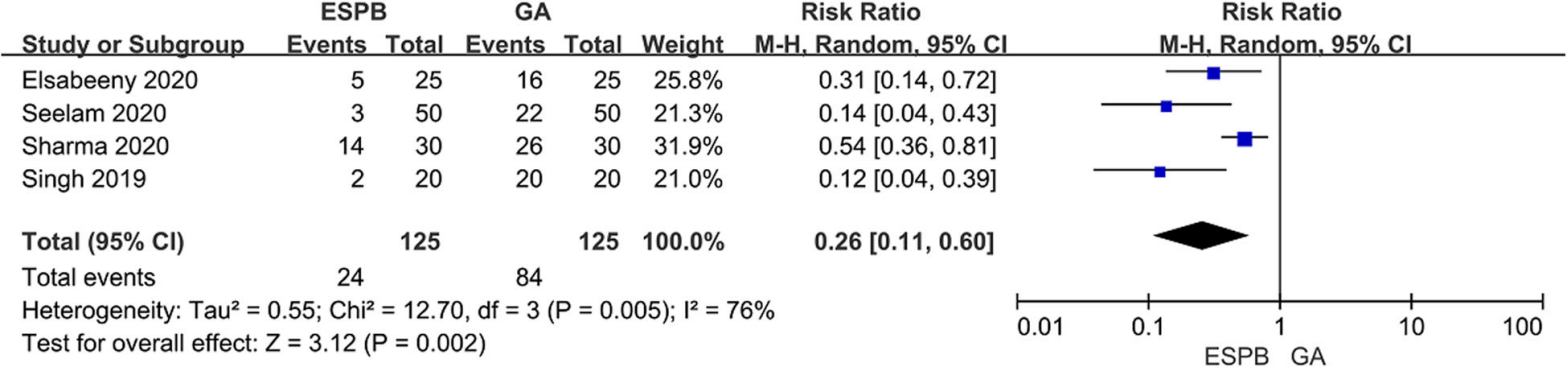
Forest plot of postoperative rescue analgesia

Four studies [17, 22–24] included 250 patients reported postoperative rescue analgesia. The number of patients who received rescue analgesia in the ESPB group was significantly lower than that in GA group (RR = 0.26, 95% CI [0.11to 0.60], *P* = 0.002) (Fig. 8).

### Incidence of complication associated with the nerve block

There were no complications such as local anesthetic toxicity, pneumothorax, vascular puncture or respiratory depression associated with ESPB were reported in all studies that included 679 patients. One study reported skin itch caused by morphine [25].

### Publication bias

Visual inspection of the funnel plot for postoperative opioid consumption showed considerable asymmetry and the Egger’s test showed a significant result, indicating the presence of a potential publication bias (*P* = 0.007) [see Additional Fig.S2]. The funnel plot for incidence of PONV showed slight asymmetry, while no significant publication bias was observed on Egger’s test (*P* = 0.193) [see Additional Fig.S3].

## Discussion

Our meta-analysis demonstrated the clinical analgesic efficacy of erector spinae plane block in patients after breast cancer surgery. Specifically, for opioid consumption at 24 h postoperatively, we found ESPB to be superior to Control by clinically important differences. Additionally, ESPB had a significant reduction in pain scores (VAS/NRS) at the first 24 h after surgery. Furthermore, ESPB was more beneficial to decrease intraoperative opioid consumption, incidence of PONV, as well as the need for rescue analgesia. There was no statistically significant difference about complications after surgery related to ESPB. These results support the analgesic utility of erector spinae plane block in patients having breast cancer surgery.

Erector spinae plane block is an ultrasound-guided novel interfascial plane block where local anesthetic is injected to the plane between thoracic transverse process and erector spinae muscle. The mechanism of ESPB was thought to be similar to paravertebral block, which achieve a multi-dermatomal sensory block of the posterior, lateral, and anterior thoracic wall [8]. This notion is further strengthened by the cadaver studies of Chin et al. [27] and Yang et al. [28] and the clinical studies of Ueshima et al. [29]. However, another potential mechanism of action is likely linked to the epidural spread as some studies have been found bilateral sensory block caused by unilateral ESPB [30–34].

Several studies have explored the feasibility of ESPB in improving postoperative pain after breast surgery. One recent systematic review reported that ESPB could decrease postoperative pain and opioid consumption when used as a part of multimodal analgesia in patients after breast surgery [35]. The results of our meta-analysis showed that, except for 1 h after surgery, ESPB combined with GA significantly reduced opioid consumption 6-24 h after breast surgery compared with GA alone. One possible reason for this is the early postoperative analgesic effects of intraoperative opioids and nonsteroidal anti-inflammatory drugs. However, we need to be cautious about the above results due to the considerable heterogeneity of the results and the limited number of trials that met the inclusion criteria.

Our meta-analysis showed that ESPB reduced VAS pain score by 1.02 points 1 h and 0.59 points 24 h after surgery, respectively. Furthermore, the reduction in pain score within 24 h after surgery decreased as time went on. It may be related to the weakening of local anesthetic effect by the reduction of concentration after metabolism. Although some may question the clinical significance of a 0.59 reduction in the pain score. However, while achieving this goal, the number of patients requiring postoperative analgesia and the consumption of opioids during and after surgery are reduced at the same time, rather than relying on a large number of opioids to reduce the pain score. The above results confirm the analgesic effect of ESPB in modified radical mastectomy.

There are a very limited number of articles reported complications related to ESPB, two studies have reported pneumothorax associated with ESPB [36, 37]. In the current meta-analysis, we found no statistically significant difference in complications associated with ESPB probably due to the site of injection is far from the pleura and major blood vessels. Moreover, the incidence of PONV in the current study was significantly lower in patients receiving ESPB than GA alone, the decrease of opioid use after ESPB may have contributed to reduce PONV incidence in these patients.

Despite strict inclusion and exclusion criteria to standardize the included studies, there was still a high heterogeneity in this meta-analysis. However, the exclusion of each study did not change the final conclusions drawn from the pooled analysis. A major factor contributing to heterogeneity is the different surgical methods and the diversity of postoperative analgesia. Although opioids were converted to equivalent doses just as other studies did [38], supplementary analgesics such as tramadol and paracetamol were used in some trials, making it more difficult to compare the opioids among trials. Another influencing factor about heterogeneity might be the differences of selection, dosage and concentration of local anesthetic used in each study. Although the subgroup analysis confirmed that bupivacaine was in good agreement with ropivacaine. The type and optimal dose of local anaesthetic in ESPB for breast surgery are still unknown and well-designed randomized controlled trials are still needed. One randomized controlled trial investigating bupivacaine 0.375% vs bupivacaine 0.25% in ESP block showed that the higher concentration of bupivacaine significantly reduced the use of tramadol after radical breast cancer surgery [39].

Although the above factors contributed to the heterogeneity of the results, we failed to change it. Our meta-analysis also has some other limitations. First, the results of postoperative opioid consumption indicated the presence of a potential publication bias, while the results of nausea and vomiting indicated no publication bias, which may be related to the small number of included studies and the small sample size of the literature. The results of the relationship between ESPB and postoperative opioid consumption could be overestimated. Second, very few trials assessed sensory testing for mapping the block area [23–25] that some trials could not evaluate the efficacy of the ESP block, it would be better to show the extent of the blockage for further comparison. Finally, this meta-analysis is unable to estimate the incidence rate of complication accurately because rare events require a larger sample size. Despite the above-mentioned limitations, the current study is still most updated and comprehensive meta-analysis.

## Conclusions

In conclusion, our meta-analysis revealed that ultrasound-guided ESPB provided better postoperative pain control by reducing perioperative opioid consumption and VAS pain scores in patients after breast cancer surgery, in comparison to GA alone. Perioperative ESPB can be a feasible technique in the field of breast surgery for multimodal analgesia. Our results, however, should be interpreted cautiously because of the high levels of heterogeneity, more large-sample and high-quality RCTs are required to verify and strengthen our results.

## Supplementary Information

**Additional file 1.** The full search terms for each database.

**Additional file 2 Fig. S1**. A subgroup analysis of low-risk of bias studies versus some concerns of bias studies.

**Additional file 3 Fig. S2**. The funnel plot and Egger’s test for postoperative opioid consumption at the first 24 h after surgery.

**Additional file 4 Fig. S3**. The funnel plot and Egger’s test for incidence of PONV.

## Data Availability

All data generated and analyzed during this study are included within this published article and its supplementary information files.

## Abbreviations

ESPB: erector spinae plane block
RCTs: randomized controlled trials
GA: general anesthesia
PONV: postoperative nausea and vomiting
MD: mean difference
CI: confidence interval
