# Supplementary material for: Analgesic efficacy and safety of erector spinae plane block in breast cancer surgery: a systematic review and meta-analysis
Source: BMC Anesthesiol. 2021 Feb 20;21:59. doi: 10.1186/s12871-021-01277-x (PMC7896394; doi:10.1186/s12871-021-01277-x)
Supplement: Supplementary file 1 — Additional file 1. The full search terms for each database. [file 12871_2021_1277_MOESM1_ESM.docx]

**Analgesic efficacy and safety of erector spinae plane block** **in breast cancer surgery**

**A systematic review and meta-analysis**

Ying Zhang^1^, Tieshuai Liu^1^, Youfa Zhou^1^, Yijin Yu^1^, Gang Chen^1*^

^1^ Department of Anesthesiology, Sir Run Run Shaw Hospital, School of Medicine, Zhejiang University, Hangzhou 310020, China.

*Correspondence: Gang Chen

*Email: chengang120@zju.edu.cn

*Department of Anesthesiology, Sir Run Run Shaw Hospital, School of Medicine, Zhejiang University, Hangzhou 310020, China.

**Appendix –Embase Search Strategy**

#1 'breast tumor'/exp 574075

#2 'breast neoplasms':ab,ti 955

#3 'neoplasm, breast':ab,ti 20

#4 'tumor, breast':ab,ti 390

#5 'breast cancer':ab,ti 406583

#6 'cancer, breast':ab,ti 3396

#7 'cancer of breast':ab,ti 157

#8 'mammary cancer':ab,ti 4042

#9 'cancer, mammary':ab,ti 84

#10 'breast carcinoma':ab,ti 32498

#11 'carcinoma, breast':ab,ti 1104

#12 'human mammary carcinomas':ab,ti 173

#13 'human mammary neoplasms':ab,ti 3

#14 'mastectomy'/exp 63889

#15 'mammectomy':ab,ti 110

#16 'mastectomies':ab,ti 2490

#17 'mammectomies':ab,ti 8

#18 'breast surgery':ab,ti 6359

#19 'breast cancer surgery':ab,ti 3647

#20 #1 OR #2 OR #3 OR #4 OR #5 OR #6 OR #7 OR #8 OR #9 OR #10 OR #11 OR #12 OR #13 OR #14 OR #15 OR #16 OR #17 OR #18 OR #19 628119

#21 'erector':ab,ti 3530

#22 'erector spinae':ab,ti 3179

#23 'erector spinae block':ab,ti 77

#24 'erector spinae plane block':ab,ti 591

#25 'esp':ab,ti 5065

#26 'esp block':ab,ti 181

#27 'espb':ab,ti 410

#28 #21 OR #22 OR #23 OR #24 OR #25 OR #26 OR #27 8629

#29 'randomized controlled trial':ab,ti 103337

#30 'random*':ab,ti 1605639

#31 'placebo':ab,ti 316412

#32 'double-blind':ab,ti 199942

#33 #29 OR #30 OR #31 OR #32 1735152

#34 #20 AND #28 AND #33 44

**Appendix –Pubmed Search Strategy**

1. "Breast Neoplasms"[Mesh] 297,759

2. (((((((((((Neoplasm, Breast[Title/Abstract]) OR (Breast Tumors[Title/Abstract])) OR (Tumor, Breast[Title/Abstract])) OR (Breast Cancer[Title/Abstract])) OR (Cancer, Breast[Title/Abstract])) OR (Cancer of Breast[Title/Abstract])) OR (Mammary Cancer[Title/Abstract])) OR (Cancer, Mammary[Title/Abstract])) OR (Breast Carcinoma[Title/Abstract])) OR (Carcinoma, Breast[Title/Abstract])) OR (Human Mammary Carcinomas[Title/Abstract])) OR (Human Mammary Neoplasms[Title/Abstract]) 301,597

3. "Mastectomy"[Mesh] 32,136

4. ((((Mammectomy[Title/Abstract]) OR (Mastectomies[Title/Abstract])) OR (Mammectomies[Title/Abstract])) OR (breast surgery[Title/Abstract])) OR (breast cancer surgery[Title/Abstract]) 7,770

5. 1 or 2 or 3 or 4

6. ((((((erector[Title/Abstract]) OR (erector spinae[Title/Abstract])) OR (erector spinae block[Title/Abstract])) OR (erector spinae plane block[Title/Abstract])) OR (esp[Title/Abstract])) OR (ESP block[Title/Abstract])) OR (ESPB[Title/Abstract]) 6,319

7. (((((randomized controlled trial[Publication Type]) OR (controlled clinical trial[Publication Type])) OR (randomized[Title/Abstract])) OR (placebo[Title/Abstract])) OR (random[Title/Abstract])) OR (controlled[Title/Abstract]) 1,648,636

8. 5 and 6 and 7 21

("Breast Neoplasms"[MeSH Terms] OR ("neoplasm breast"[Title/Abstract] OR "breast tumors"[Title/Abstract] OR "tumor breast"[Title/Abstract] OR "breast cancer"[Title/Abstract] OR "cancer breast"[Title/Abstract] OR "cancer of breast"[Title/Abstract] OR "mammary cancer"[Title/Abstract] OR "cancer mammary"[Title/Abstract] OR "breast carcinoma"[Title/Abstract] OR "carcinoma breast"[Title/Abstract] OR "human mammary carcinomas"[Title/Abstract] OR "human mammary neoplasms"[Title/Abstract]) OR "Mastectomy"[MeSH Terms] OR ("Mammectomy"[Title/Abstract] OR "Mastectomies"[Title/Abstract] OR "Mammectomies"[Title/Abstract] OR "breast surgery"[Title/Abstract] OR "breast cancer surgery"[Title/Abstract])) AND ("erector"[Title/Abstract] OR "erector spinae"[Title/Abstract] OR "erector spinae block"[Title/Abstract] OR "erector spinae plane block"[Title/Abstract] OR "ESP"[Title/Abstract] OR "esp block"[Title/Abstract] OR "ESPB"[Title/Abstract]) AND ("randomized controlled trial"[Publication Type] OR "controlled clinical trial"[Publication Type] OR "randomized"[Title/Abstract] OR "placebo"[Title/Abstract] OR "random"[Title/Abstract] OR "controlled"[Title/Abstract])

**Appendix –web of science Search Strategy**

TS=(Breast Neoplasms or Neoplasm, Breast or Breast Tumors or Tumor, Breast or Breast Cancer or Cancer, Breast or Cancer of Breast or Mammary Cancer or Cancer, Mammary or Breast Carcinoma or Carcinoma, Breast or Human Mammary Carcinomas or Human Mammary or Neoplasms or Mastectomy or Mammectomy or Mastectomies or Mammectomies or breast surgery or breast cancer surgery)

TS=(Erector or erector spinae or erector spinae block or erector spinae plane block or ESP block or ESPB or esp)

TS=(random*controlled trial or random*)

**Appendix –Cochrane library Search Strategy**

#1 MeSH descriptor: [Breast Neoplasms] explode all trees

#2 (Neoplasm, Breast):ti,ab,kw OR (Breast Tumors):ti,ab,kw OR (Tumor, Breast):ti,ab,kw OR (Breast Cancer):ti,ab,kw OR (Cancer, Breast):ti,ab,kw

#3 (Cancer of Breast):ti,ab,kw OR (Mammary Cancer):ti,ab,kw OR (Cancer, Mammary):ti,ab,kw OR (Breast Carcinoma):ti,ab,kw OR (Carcinoma, Breast):ti,ab,kw

#4 (Human Mammary Carcinomas):ti,ab,kw OR (Human Mammary Neoplasms):ti,ab,kw

#5 MeSH descriptor: [Mastectomy] explode all trees

#6 (Mammectomy):ti,ab,kw OR (Mastectomies):ti,ab,kw OR (Mammectomies):ti,ab,kw OR (breast surgery):ti,ab,kw OR (breast cancer surgery):ti,ab,kw

#7 #1 or #2 or #3 or #4 or #5 or #6

#8 (Erector):ti,ab,kw OR (erector spinae):ti,ab,kw OR (erector spinae block):ti,ab,kw OR (erector spinae plane block):ti,ab,kw OR (ESP block):ti,ab,kw

#9 (ESPB):ti,ab,kw OR (esp):ti,ab,kw

#10 #8 or #9

#11 #7 and #10

**Appendix –ClinicalTrials.gov**

Search the website www. ClinicalTrials.gov

1. Condition or disease: breast cancer

2. Condition or disease: mastectomy

3. Intervention/treatment: erector spinae plane block

4. Intervention/treatment: erector

5. Study Results: studies with results

(1 or 2) and (3 or 4) and 5
