# Supplementary figures and images for: Analgesic efficacy and safety of erector spinae plane block in breast cancer surgery: a systematic review and meta-analysis
Source: BMC Anesthesiol. 2021 Feb 20;21:59. doi: 10.1186/s12871-021-01277-x (PMC7896394; doi:10.1186/s12871-021-01277-x)

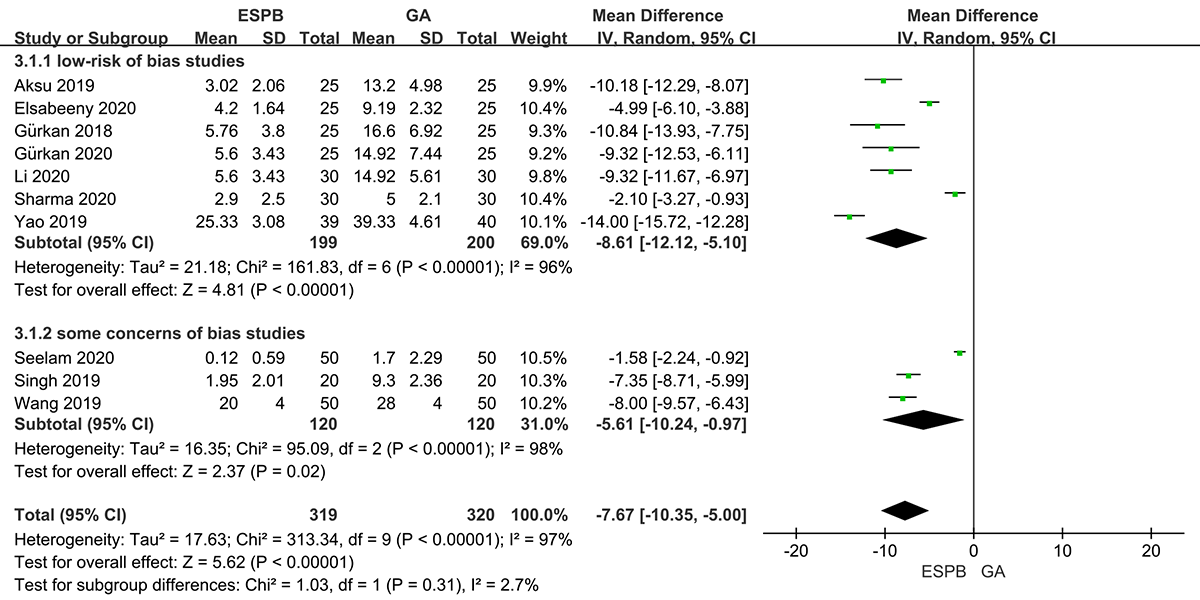

Supplement: Supplementary file 2 — Additional file 2 Fig. S1. A subgroup analysis of low-risk of bias studies versus some concerns of bias studies. [file 12871_2021_1277_MOESM2_ESM.tif]

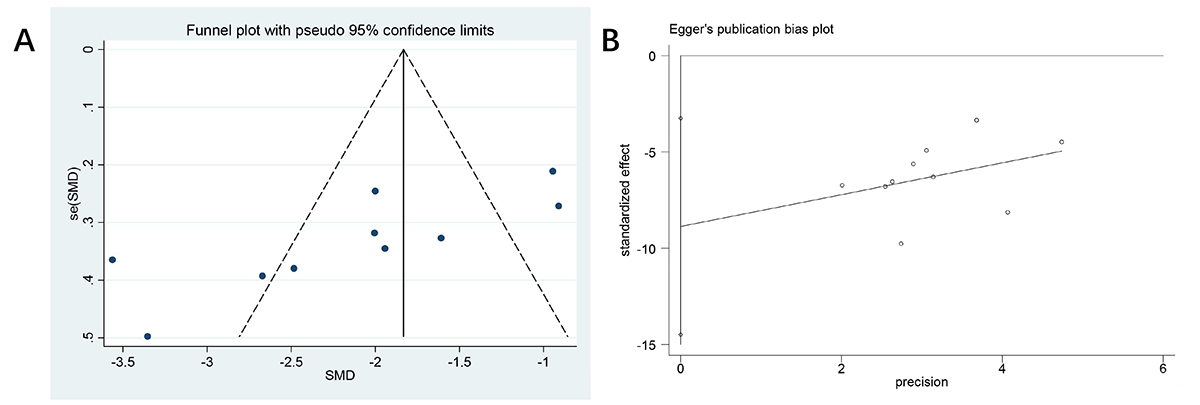

Supplement: Supplementary file 3 — Additional file 3 Fig. S2. The funnel plot and Egger’s test for postoperative opioid consumption at the first 24 h after surgery. [file 12871_2021_1277_MOESM3_ESM.tif]

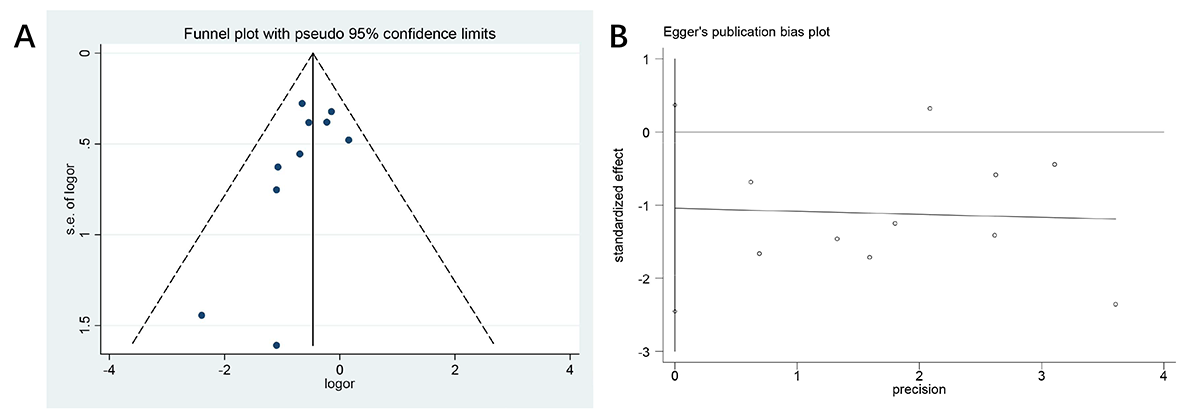

Supplement: Supplementary file 4 — Additional file 4 Fig. S3. The funnel plot and Egger’s test for incidence of PONV. [file 12871_2021_1277_MOESM4_ESM.tif]
